# Regulating Cells Fate and Function to Facilitate Bone Regeneration via Designing Programmable Bio‐Interactive Materials

**DOI:** 10.1002/advs.77087

**Published:** 2026-08-30

**Authors:** Qingrui Fan, Fuming Cao, Junqiang Mao, Pengbin Yin, Licheng Zhang, Jianjun Wang, Peifu Tang

**Affiliations:** ^1^ Department of Orthopedics Chinese PLA General Hospital Beijing People's Republic of China; ^2^ Interdisciplinary Research Center For Advanced Materials Technical Institute of Physics and Chemistry Chinese Academy of Sciences Beijing People's Republic of China; ^3^ National Clinical Research Center for Orthopedics Sports Medicine & Rehabilitation Beijing People's Republic of China

**Keywords:** bone healing, bone regeneration, bone tissue, bone tissue engineering

## Abstract

Achieving efficient bone repair necessitates a thorough understanding of the physiological activities of native bone tissue, enabling selection and design of appropriate materials. To date, artificial scaffolds have been developed from inert materials to bioactive materials and demonstrate the capabilities to promote bone repair. However, the complex physiological changes that occur during bone repair pose significant challenges to their practical clinical applications. In this Review, we summarize the studies of bone biology and the evolution of materials design in the context of bone‐tissue engineering applications. Specifically, we emphasize the role of biomaterials from a materials science perspective in regulating cell fates. Furthermore, we introduce the concept of programmable bio‐interactive materials as a potential solution for bone regeneration. These materials possess the ability to dynamically and precisely interact with biological systems, guiding the bone‐tissue regeneration process. Additionally, we discuss the unmet needs and current challenges faced in the development of bio‐interactive materials for bone‐tissue regeneration. By highlighting emerging strategies in the field, we aim to shed light on future directions for research and development in this exciting area of study.

## Graphical abstract

This article aims to elucidate the biological mechanisms of bone repair and the evolution of material design, highlighting key cellular and molecular processes. It further proposes strategies and prospects for programmable bio‐interactive materials, which enable precisely guided bone tissue regeneration by dynamically regulating cell behavior and the microenvironment to enhance therapeutic outcomes.

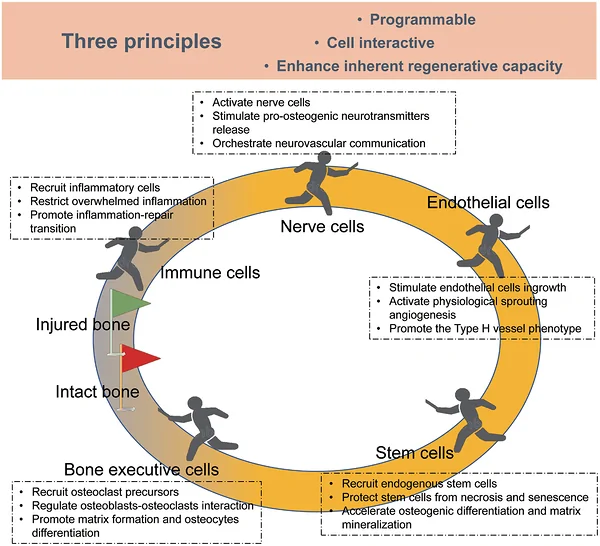

## 1 Introduction

Bone defects are a major global public health concern with significant economic implications. They not only impact individuals' overall well‐being and quality of life but also result in work absenteeism, high healthcare costs, and burden on families, societies, and healthcare systems [1, 2]. Annually, over 20 million people worldwide require regenerative dental or orthopedic treatments, and the cost of treating bone problems in Europe alone ranges from 10 000 to 100 000 Euros.

Age‐related fractures are expected to increase in the United States from 2.1 million in 2005 to over 3 million fractures in 2025, solely considering the elderly/aging population at risk. In Europe, the annual number of fractures is estimated to rise 28% from 2010 to 2025, with an absolute increase from 3.5 million to 4.5 million injuries, respectively [3].

Current treatments, such as autografts or alrafts/xenografts, have limitations including donor‐site morbidity and graft rejection. Consequently, extensive research and development of artificial scaffolds for bone repair have been conducted [4]. These artificial scaffolds have progressed from simply inert materials to biologically active components, incorporating cells and growth factors to facilitate bone regeneration [5, 6]. Nevertheless, the effectiveness of designed scaffolds in clinical applications has been restricted [7].

Traditional tissue engineering approaches have attempted to incorporate biological activity to promote bone regeneration but have been poorly executed for several reasons [4, 8]. First, they lack temporal and spatial characteristics; the addition of cells or growth factors only satisfies the needs of regeneration at a specific moment, whereas an in vivo bone repair process is dynamic and constantly changing. Existing tissue engineering technologies usually fail to meet these spatio‐temporal requirements. Second, these exogenous bioactive factors have potential immune responses and the risks of disease transmission; therefore, their biosafety still needs more stringent basic and clinical verification. Third, current bone tissue scaffolds primarily serve as mechanical support and loading platforms for bioactive factors, while disregarding the significance of the scaffolds themselves in regulating cell fates. For example, the properties of scaffold materials usually lack endogenous regulation and do not resemble an extracellular matrix to support the body's own cell activities, including stem cell recruitment, proliferation, and differentiation for bone repair. However, endogenous cells play a critical role in actual repair processes, such as the body's own cells will secret cytokines and growth factors, which are necessary for bone repair. Lastly, traditional tissue engineering approaches have poor responsiveness and control, often leading to issues such as non‐degradable foreign bodies. Therefore, ideal materials should be able to respond to signals from the body and provide feedback‐based intervention to supplement endogenous deficiencies or eliminate factors that hinder bone regeneration, providing necessary supplementation and assistance to enhance bone regeneration.

It is therefore imperative that future materials for bone regeneration be programmable and bio‐interactive. Programmability would allow these materials to be designed and controlled for specific functions and tasks, enabling customization and adaptation to different regenerative processes. Bio‐interactivity would ensure active interaction with biological systems, including communication and response to signals from the body, facilitating dynamic interaction with surrounding cells and tissues. This bio‐interactivity would actively promote and enhance the process of bone regeneration. The goal is to develop materials that go beyond the passive nature of traditional materials used in bone tissue engineering, possessing the ability to be programmed and interact dynamically with the biological environment, ultimately enhancing the regenerative process.

At the dawn of the profound conceptual transformation, we believe it is necessary to conduct a comprehensive review to summarize recent advancements of design and application in bone scaffold materials, define programmable bio‐interactive materials, classify how bio‐interactive materials affect the fate and function of cells to promote bone regeneration from the perspective of immunoregulation, osteovascularization, and neuroization. Such a review would provide valuable guidance for the development of next‐generation bone repair materials, further advancing the field of bone‐tissue engineering.

## 2 Bone Defects

**FIGURE 1:**
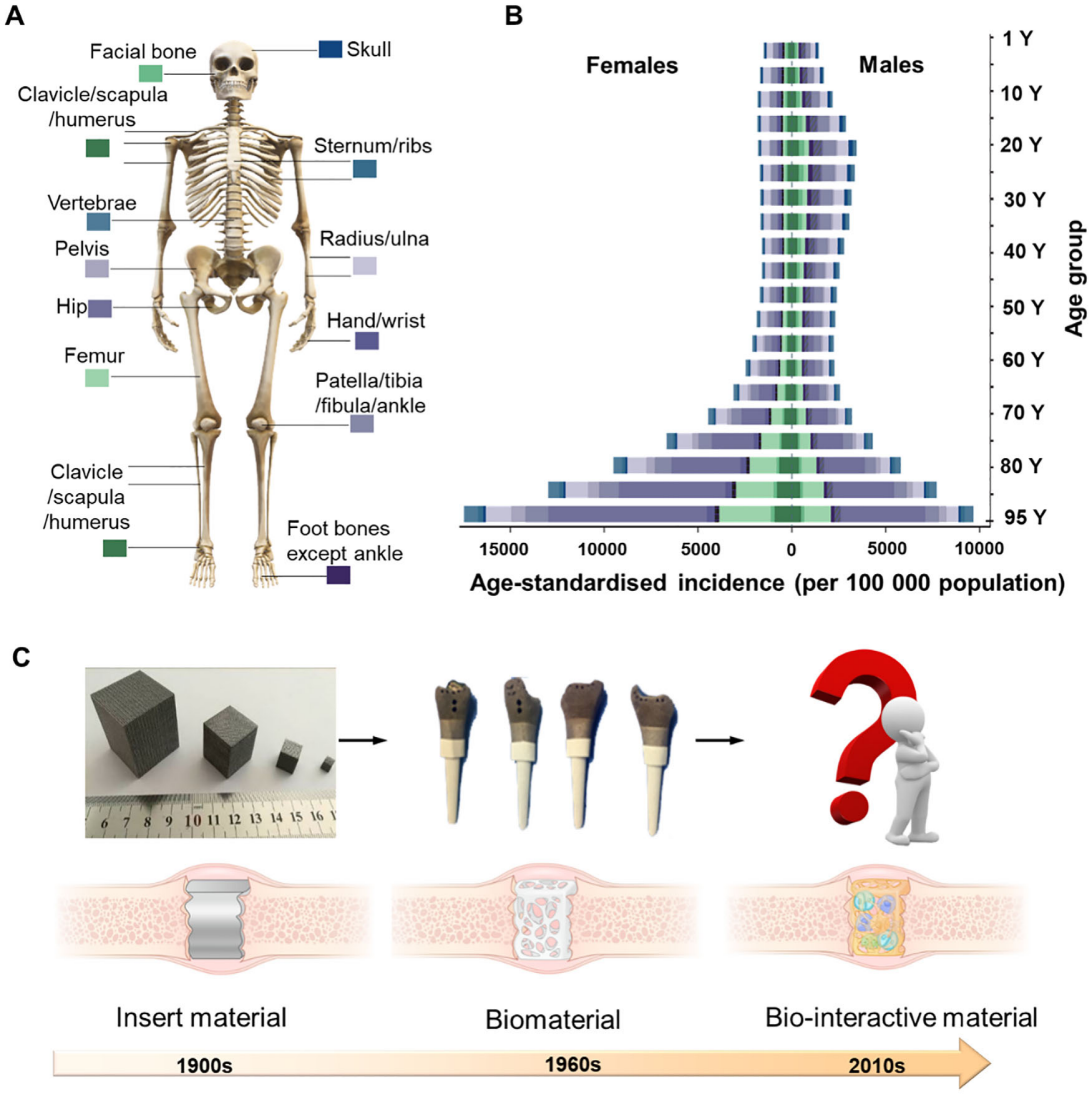
(A) Human skeletal system and common sites of bone injury. (B) Age‐specific incidence rate of fractures for each anatomical site globally, by age group and sex, data from the Global Burden of Disease Study 2019 [9]. (C) Timeline of major milestones in materials design for bone‐tissue engineering. Initial efforts toward bone repair involved the use of resorbable metals and calcium phosphates (early 20th century). Since the observation that bone can form in Bioglass (the first human‐made material capable of bonding to living tissues, developed in the 1960s), and by using growth factors and stem cells, the field of bone‐tissue engineering has emerged as a separate research area.

Bone defects are significant health issues that can occur due to external injuries or internal diseases. While bones have a natural ability to regenerate and heal small damages, large bone defects exceeding a critical size threshold (typically over 2 cm) require clinical intervention for complete healing [8, 10]. Common sites of bone defects include the skull, facial bones, sternum, ribs, clavicle, scapula, humerus, radius, ulna, hand, wrist, vertebral column, pelvis, hip, femur, patella, tibia, fibula, and foot bones, as illustrated in Figure 1A [9, 11, 12]. These defects can result from various causes, such as traumatic injuries, degenerative diseases, congenital defects, or surgical removal of tumors. It is essential to address these bone defects as they can impact the overall mechanical support, organ protection, and metabolic functions provided by the skeletal system. Research and clinical practices focus on finding effective solutions for bone repair in these cases. Age‐specific incidence rates of bone defects are measured per 100 000 individuals for each anatomical site globally (Figure 1B). These data allow researchers and healthcare professionals to analyze the prevalence of bone defects based on age groups, sex, and anatomical locations using a standardized population structure [2, 9].

## 3 Timeline of Major Milestones in Scaffolds for Bone‐Tissue Engineering

The history of bone‐tissue engineering scaffolds can be traced back thousands of years, when people in ancient Egypt used wood to fill bone defects [13, 14, 15]. In modern medicine, the development of bone defect repair began in the 1800s. Initially, metals and calcium phosphates were used as implant materials due to their favorable mechanical properties. (Figure 1C) [16]. Metal‐based implants, such as bio‐metals and alloys, are still commonly used in clinical practice today. However, the release of toxic metallic elements through corrosion can lead to adverse tissue reactions and hypersensitivity. Additionally, the mechanical properties of metals may not match those of natural bone, potentially resulting in stress shielding and loss of normal bone tissue function [14]. As materials synthesis techniques and processing technologies have advanced, it has become possible to fabricate bone implants with tunable physicochemical properties. Biomaterials such as bio‐glass and polymer composites have been developed using methods like electrospinning and 3D printing [6, 17, 18]. Meanwhile, advances in our understanding of the physiological characteristics of bone tissue, including its hierarchical structure, cells, growth factors, and cytokines [19, 20, 21, 22, 23, 24, 25], have provided insights for biomimetic design of bone‐regenerative biomaterials. While some biomaterials have been successfully commercialized for clinical use, there is ongoing research in the field of bio‐interactive materials. These materials focus on utilizing growth factors and stem cells to promote bone regeneration, and they are an emerging area of study within bone‐tissue engineering [26, 27, 28].

## 4 Self‐Healing of Injured Bone Tissue

When it comes to minor bone injuries, the bone has the capacity for self‐healing through two primary pathways: intramembranous bone formation and endochondral bone formation [3, 29].

**FIGURE 2:**
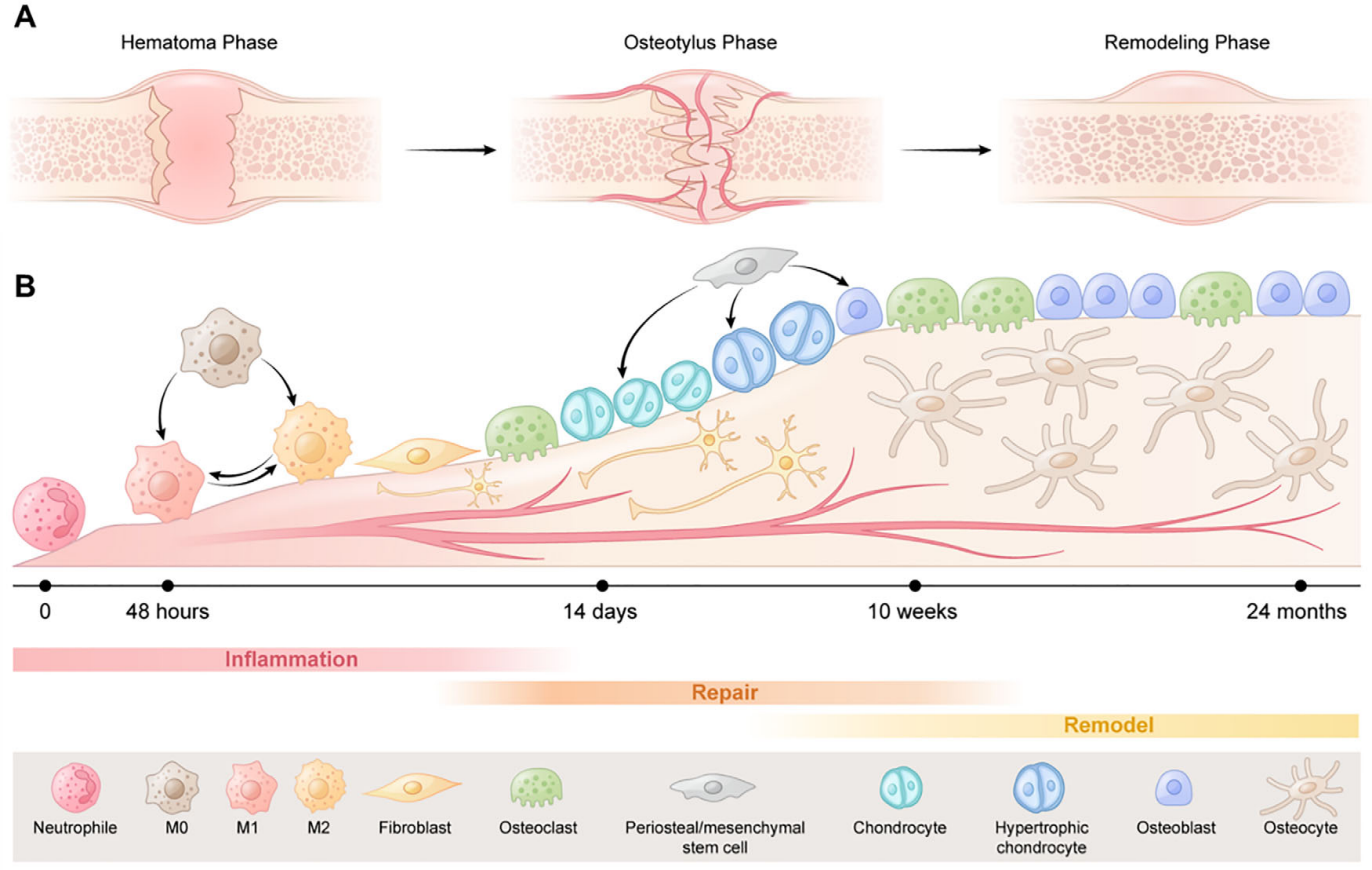
The brief process of physiological activity for self‐healing bone tissue. (A) Based on the type of injury the self‐healing process can be divided into three main biological stages: hematoma, osteotylus, and remodeling phase. (B) Schematic representation of the typical bone cells involved in bone regeneration, and the time span of their prevalence in each stage, are denoted, and their functions will be discussed in the main text.

In the intramembranous ossification process, mesenchymal stem cells (MSCs) present in the mesenchyme or medullary cavity directly differentiate into osteoblasts. This pathway does not involve blood vessel formation and typically leads to limited‐area bone regeneration. Detailed discussions regarding intramembranous ossification can be found in excellent review articles beyond the scope of our present discussion (literature references available upon request) [3]. In most clinical bone injuries, endochondral bone formation plays a crucial role as it has the inherent ability to form vascularized bone [30]. The process of endochondral ossification‐driven bone regeneration can be divided into three phases: the hematoma (inflammation) phase, the osteoblasts phase, and the bone remodeling phase (Figure 2A) [31]. Importantly, intramembranous ossification and endochondral ossification both play critical and complementary roles in bone healing. These two modes of bone formation are not mutually exclusive but rather operate in a coordinated spatiotemporal manner to ensure successful bone regeneration [32, 33]. Typically, the intensity of stimulation determines whether bone formation occurs through the intramembranous pathway or the endochondral pathway: intramembranous ossification occurs predominantly in the callus region away from the fracture site, adjacent to the distal cortex and periosteal ends, where mechanical strain is relatively low. In contrast, endochondral ossification dominates the area between the fracture ends and the outer region of the periosteal callus, which is typically subjected to higher mechanical strain and lower oxygen tension [34, 35]. In the following discussion, we will explore the representative types of cells and related cytokines involved in bone regeneration. This will include an examination of the evolutionary process of inflammation and anti‐inflammation, as well as the regeneration of blood vessels, nerves, and bone (Figure 2B).

## 5 Hematoma Phase

The hematoma phase, also known as the inflammatory stage, is an initial and crucial stage in bone healing [36]. When blood vessels surrounding the injured bone rupture, the coagulation cascade system in the blood plasma is activated, leading to the formation of a hematoma [37, 38]. Simultaneously, various immune cells, such as neutrophils, mast cells, T cells, monocytes, and macrophages, are released from the blood vessels and initiate the inflammatory cascade [39]. In the following sections, we will systematically discuss the roles of neutrophils and macrophages in the hematoma phase.

### 5.1 Neutrophils

Neutrophils, derived from the bone marrow, are among the first immune cells recruited to the injury site. Their recruitment and apoptosis are regulated by granulocyte colony‐stimulating factor (G‐CSF) [40, 41, 42]. Neutrophils secrete various factors, including chemokines, cytokines, growth factors, and lipid mediators, which play a role in regulating their own activities as well as recruiting other immune cells like T cells, monocytes, and macrophages. In‐depth discussions on the roles of neutrophils can be found in relevant review articles [41, 42, 43].

### 5.2 Macrophage

Macrophages originate from either circulating monocytes or tissue‐resident macrophage populations [44]. These cells exhibit remarkable plasticity, switching phenotypes in response to local microenvironmental cues [45, 46, 47]. Classically activated M1 macrophages are driven by pro‐inflammatory stimuli such as interferon‐gamma (IFN‐γ), lipopolysaccharide (LPS), tumor necrosis factor‐alpha (TNF‐α), and macrophage colony‐stimulating factor (CSF‐1/M‐CSF). M1 macrophages dominate the injury site for 3–4 days before transitioning to the anti‐inflammatory M2 phenotype, which is induced by signals like interleukin‐4 (IL‐4) and IL‐13 from basophils, mast cells, and other granulocytes. M2 macrophages are characterized by the expression of markers such as the mannose receptor (CD206), secretion of anti‐inflammatory mediators (e.g., IL‐10), and promotion of tissue repair.

Notably, the M2 classification encompasses a spectrum of functionally distinct subsets—M2a, M2b, M2c, M2d, and M2f—each with unique roles, the details also can follow these excellent reviews [48, 49, 50, 51]: (i) M2a (IL‐4/IL‐13–activated) enhances cellular proliferation and angiogenesis via growth factors like platelet‐derived growth factor (PDGF), transforming growth factor‐β1 (TGF‐β1), insulin‐like growth factor 1 (IGF‐1), and vascular endothelial growth factor A (VEGF). (ii) M2b (induced by immune complexes and Toll‐like receptor agonists) regulates immunity through Th2 lymphocyte activation, secreting IL‐10 and IL‐6. (iii) M2c (activated by IL‐10, glucocorticoids, or TGF‐β) resolves inflammation via IL‐10 and matrix metalloproteinases (MMPs). (iv) M2d (stimulated by adenosine receptor agonists and IL‐6) supports tumor‐associated immunosuppression and angiogenesis. (v) M2f (induced by factors like pericyte‐derived signals) upregulates genes linked to pericyte differentiation.

In the context of biomaterial design, studies typically evaluate immunomodulatory performance based on the ability of materials to suppress prolonged M1 responses and promote the transition toward pro‐regenerative macrophage phenotypes [52]. However, this simplified model has important limitations for guiding next‐generation biomaterial design. First, material‐induced immune responses likely involve multiple macrophage subsets and other immune cells, which cannot be fully represented by the M1/M2 paradigm [53]. Second, macrophage phenotypes during bone healing are highly dynamic and evolve over time, suggesting that static material designs targeting only a single phenotype may not fully recapitulate the physiological healing process [54]. Third, recent single‐cell analyses indicate that macrophage functions in bone regeneration may be linked to specific transcriptional programs and tissue‐resident macrophage populations that are not captured by the traditional classification [55].

Therefore, future biomaterial strategies may need to move beyond the M1/M2 dichotomy and focus on precisely regulating macrophage functional states within a spatiotemporally controlled immune microenvironment. Integrating high‐resolution tools such as single‐cell sequencing, lineage tracing, and spatial omics with biomaterial design will likely provide deeper insight into macrophage heterogeneity and enable the development of more sophisticated immunomodulatory scaffolds.

## 6 Callus Phase

Following the hematoma phase, the healing process enters the callus phase. During this phase, the mesenchymal stem cells (MSC) that formed the fibrovascular callus differentiate into either chondrocytes or osteoblasts [56]. At this stage, the formation of hypertrophic chondrocytes is a pivotal state during endochondral ossification as it can induce vascular invasion into the cartilage callus by secreting VEGF, PDGF, and PLGF (placental growth factor), as well as stimulate the invasion of mesenchymal progenitor cells that differentiate into bone‐forming osteoblasts and of monocytes that differentiate into osteoclasts, which ultimately results in the replacement of the initial collagenous matrix (cartilage) with trabecular bone. This stage is usually reached after 8–16 weeks post‐injury, depending on factors like the type of fracture, stabilization technique, and the patient's age and health [57].

### 6.1 Revascularization

Revascularization is a critical process during bone healing as the disruption of the periosteal, cortical, and medullary vascular supply leads to cell and tissue death [58, 59, 60]. The angiogenic response, including angiogenesis (formation of new blood vessels from existing ones) and vasculogenesis (de novo formation of blood vessels from endothelial progenitor cells within the callus), is essential for supplying the callus with oxygen, nutrients, inflammatory cells, progenitor cells, and waste product removal [61, 62, 63].

Multiple growth factors, transcription factors, and cytokines are involved in revascularization during bone repair [63, 64]. The platelet‐derived growth factor (PDGF), vascular endothelial growth factor (VEGF), and hepatocyte growth factor (HGF) have significant roles in stimulating angiogenesis. Particularly, VEGF, produced by various cells in the injured bone tissue, including inflammatory cells, hypertrophic chondrocytes, MSC and osteoblasts [65], binds to VEGF receptors (VEGFR1 and VEGFR2) and activates signaling cascades that promote endothelial cell proliferation, sprouting, and recruitment of endothelial progenitor cells (EPCs) [31]. Hypoxia‐inducible factor 1‐alpha (HIF1‐alpha) is a key regulator of VEGF expression in response to low oxygen conditions [66].

The platelet‐derived growth factor (PDGF) family is renowned for its ability to promote angiogenic and osteogenic maturation [62, 67]. PDGF is a glycolytic protein produced by platelets and other cells (e.g., osteoclasts) that can stimulate the expression of VEGF, thereby promoting and stabilizing angiogenesis [68, 69]. Additionally, PDGF is also a potent chemokine that can enhance bone tissue regeneration by activating mesenchymal stem cell populations, such as fibroblasts, osteoblasts, and chondrocytes [70]. Within the PDGF family, there is a subfamily known as PDGF‐BB, which has been well documented for its capacity to directly activate endothelial progenitor cell (EPC) activity [71].

### 6.2 Nerve Regeneration

Nerve regeneration is a vital component of the bone healing process. Intrabony nerves, which exist in various regions of the skeletal system including cortical and trabecular bone, bone marrow, and periosteum, play a crucial role in regulating bone regeneration [72, 73]. These nerves secrete bioactive factors such as neurotransmitters, neuropeptides, axon guidance factors, and neurotrophins. These bioactive factors have the ability to control the activity of osteoblasts responsible for bone deposition and osteoclasts responsible for bone resorption [73, 74]. For instance, substance P (SP), which is secreted by sensory nerves, can regulate osteoblast differentiation by increasing BMP‐2 secretion and cyclic adenosine monophosphate (cAMP) production [75, 76, 77]. Nerve growth factor (NGF) also plays a role in bone remodeling by enhancing the differentiation and growth of osteoblasts and osteoclasts through specific receptor interactions, such as tropomyosin receptor kinase A (TrkA) and low‐affinity p75 neurotrophin receptor (p75NTR) [78, 79]. These signals help optimize bone formation processes and enhance overall bone regeneration.

### 6.3 MSCs

Mesenchymal stromal cells (MSCs) are another critical player in bone defect repair. They are sourced from the periosteum and bone marrow and have a significant impact on bone healing. The activation of MSCs in bone defect repair is regulated by signals released from the defect site, such as stromal cell‐derived factor‐1 (SDF1) or CXCL12, and the notch signaling pathways. These signals promote the proliferation and migration of mesenchymal progenitor cells, contributing to bone healing [80]. The decision of progenitor cells to differentiate into chondrocytes or osteoblasts in the callus phase of healing is governed by various factors. Mechanical factors and oxygen tension are crucial in regulating cell fate, and the local microenvironment has a significant impact on chondrogenesis and osteoblastogenesis [81]. Oxygen tension, in particular, has been extensively studied and has shown that hypoxia promotes chondrogenic phenotypes [82], while higher oxygen levels promote differentiation of MSCs into osteoblasts [81, 83, 84]. The secreted growth factors also play a direct role in influencing the differentiation of MSCs [24, 85]. For instance, bone morphogenetic proteins (BMPs), which are known for their osteogenic properties, stimulate the osteoblast differentiation of MSCs by activating specific transcription factors like Runx2 and Sp7 (Osterix). BMPs also have an impact on chondrocyte differentiation [86].

## 7 Bone Remodeling Phase

The remodeling of the bony callus is an essential stage in the repair of bone defects and can take several months to years to complete. During this stage, the provisional woven bone that was initially formed needs to be degraded and replaced with mature lamellar bone. Failure to properly remodel the bone can lead to the formation of nonunion. The remodeling process is primarily driven by osteoclasts, osteoblasts, and osteocytes [31]. The osteoclasts, osteoblasts, and osteocytes would dominate the remodeling stage. Bone remodeling is a vital metabolic process that regulates bone structure and function throughout adult life, with the osteoclasts playing a key role. Imbalances in the remodeling process can result in significant disruptions in skeletal structure and function, which can lead to morbidity and a shortened lifespan [87].

### 7.1 Osteoblast

Osteoblasts, known as the chief bone‐making cells, are responsible for the production of various extracellular proteins, including osteocalcin, alkaline phosphatase, and a significant amount of type I collagen, to synthesize new bone matrix [88]. These cells originate from the bone marrow or periosteum, which contains bone marrow stromal cells (BMSCs) or periosteal stem cells (PSCs). The differentiation of osteoblasts is regulated by growth factors, cytokines, hormones, and genes. Notably, bone morphogenetic proteins (BMPs) are extensively studied growth factors that can influence the proliferation and differentiation of osteoprogenitor cells [89, 90, 91].

### 7.2 Osteoclast

Osteoclasts originate from hematopoietic monocyte/macrophage lineage precursors [20]. The nuclear factor kappa‐β ligand (RANKL) and macrophage colony stimulating factor (M‐CSF) are required to stimulate osteoclasts’ proliferation, differentiation, and survival [92, 93, 94, 95]. Mature osteoclasts are capable of secreting a diverse array of factors to facilitate bone resorption. One such factor is the secretion of hydrogen ions, which serve to dissolve the hydroxyapatite crystals that make up the mineral component of bone. Additionally, they also produce cathepsin K, an enzyme responsible for the digestion of the insoluble fraction of the matrix (primarily type I collagen) [96, 97]. Osteoclasts contribute to bone defect repair in two distinct stages. In the inflammatory phase, they break down and absorb smaller bone fragments and promote blood vessel formation. Then, near the end of the healing process, osteoclasts remodel the hard callus (woven bone) and restore the bone to its pre‐injury dimensions [96].

### 7.3 Osteocyte

Osteocytes, which originate from osteoblasts, play a crucial role in regulating bone repair by sensing mechanical stimuli. They secrete soluble factors, such as phosphate‐regulating neutral endopeptidase on chromosome X (PHEX), that are involved in bone skeletal metabolism. Osteocytes are responsible for regulating local and systemic mineralization, influencing bone formation by osteoblasts and bone resorption by osteoclasts, and sensing mechanical loads on bone [21, 22].

## 8 Strategies of Macrophage Polarization

**FIGURE 3:**
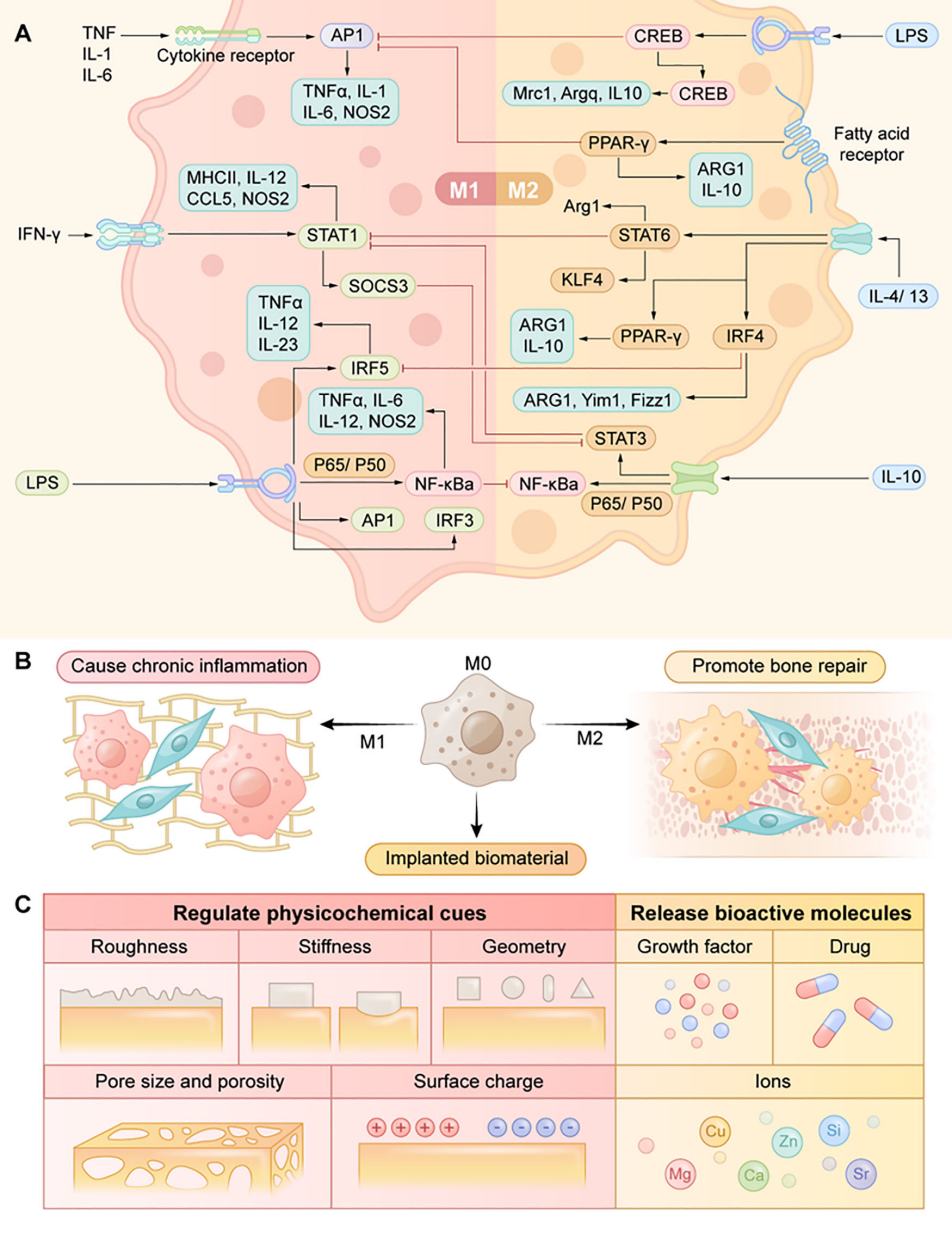
Schematic illustration of material‐modulated macrophage fate strategy to accelerate bone regeneration. (A) Mechanisms of macrophage polarization. The major pathways of macrophage polarization are outlined, and the crosstalk between the M1‐M2 macrophage–polarizing pathways is also indicated. (B) Schematic representing the macrophage inflammatory response that occurs after material implantation. Left panel: the scaffold induces macrophages to become M1, promoting a chronic inflammatory response that ultimately leads to the failure of bone biomaterials and fibrous encapsulation. Right panel: macrophages are activated into M2 subtypes, which can eliminate the inflammatory response and promote bone regeneration. (C) Schematic illustration of material‐mediated immunomodulation of macrophage fate for promoting bone regeneration. Physicochemical cues of biomaterials, such as topography, stiffness, geometry, porosity and pore size, and surface charge, can regulate macrophage polarization (M0 to M1 or M0 to M2). Releasing bioactive molecules, such as cytokines, drugs, and bioactive ions, mediate the immunomodulation.

The different forms of macrophage activation are regulated by a complex network of signaling molecules, transcription factors, epigenetic mechanisms, and posttranscriptional regulators [98, 99]. The crosstalk between the M1‐M2 macrophage–polarizing pathways is illustrated in Figure 3A. The balance between the activation of certain signaling pathways, such as STAT1, STAT3, and STAT6, determines the polarization and activity of macrophages. Activation of NF‐κB and STAT1 promotes the polarization of M1 macrophages, leading to cytotoxic and inflammatory functions. On the other hand, activation of STAT3 and STAT6 promotes the polarization of M2 macrophages, which are associated with immune suppression, tumor progression, and specific metabolic properties. Transcription factors like PPAR‐γ, PPAR‐δ, KLF4, and c‐Myc also play roles in regulating M2 macrophage function. Additionally, interleukin families are also important factors in regulating macrophage polarization, such as, inflammatory cytokines interleukin‐1 (IL‐1) and IL‐6 can enhance M1 macrophages activity the IL‐4 and IL‐10 can activate anti‐inflammatory M2 macrophages and inhibit M1 polarization [100].

The implanted biomaterials would trigger a sequential foreign body reaction initially, including nonspecific protein adsorption and fates of immune cells [101, 102, 103]. Monocytes/macrophages are among the immune cells that interact with biomaterials early on after implantation and play a crucial role in the host foreign body response to the implants. Activated macrophages secrete cytokines and chemokines that recruit other immune cells and regulate inflammation, ultimately influencing the process of bone regeneration [36, 51, 104, 105]. As discussed in Figure 2, regulating the polarization of macrophages is vital for successful bone repair, and the host response, particularly the inflammatory events at the host‐implant interface, can determine the effectiveness of implanted scaffolds in vivo [105]. To develop desired scaffolds for bone repair, the comprehension of the relationships between macrophages and scaffold materials is of paramount importance, as it can facilitate the generation of promising strategies for the fabrication of immunomodulatory biomaterials to effectively regulate the fates of macrophages [40, 106]. In this section, we discussed how artificial scaffolds regulate the fates of immune cells, including (i) physicochemical properties of the scaffolds, e.g., porosity and pore size, topography, and stiffness; (ii) releasing bioactive factors from the scaffold, e.g., growth factors, cytokines, and bioactive ions, as shown in Figure 3C.

## 9 Physicochemical Cues

Macrophage cells can sense and respond to the physical properties of underlying scaffolds. Mechanical cues, such as stiffness, surface topography, and geometrical changes, can elicit changes in macrophage elasticity and shape. These physical signals can be translated into biochemical signals that influence macrophage phenotype [107, 108].

### 9.1 Stiffness

Matrix stiffness has been found to impact macrophage fate and functions. Immune cells are capable of sensing the stiffness of their surrounding matrix and translating mechanical cues into physiological responses [109]. Studies have examined the response of macrophages to substrates with varying stiffness, ranging from 1 kPa to 100s kPa. These investigations have concluded that substrates with lower mechanical properties (<5 kPa) tend to promote an anti‐inflammatory phenotype [110]. A study also shows that electrospun fibers with low stiffness can promote macrophage differentiation toward the M2 phenotype [111].

**FIGURE 4:**
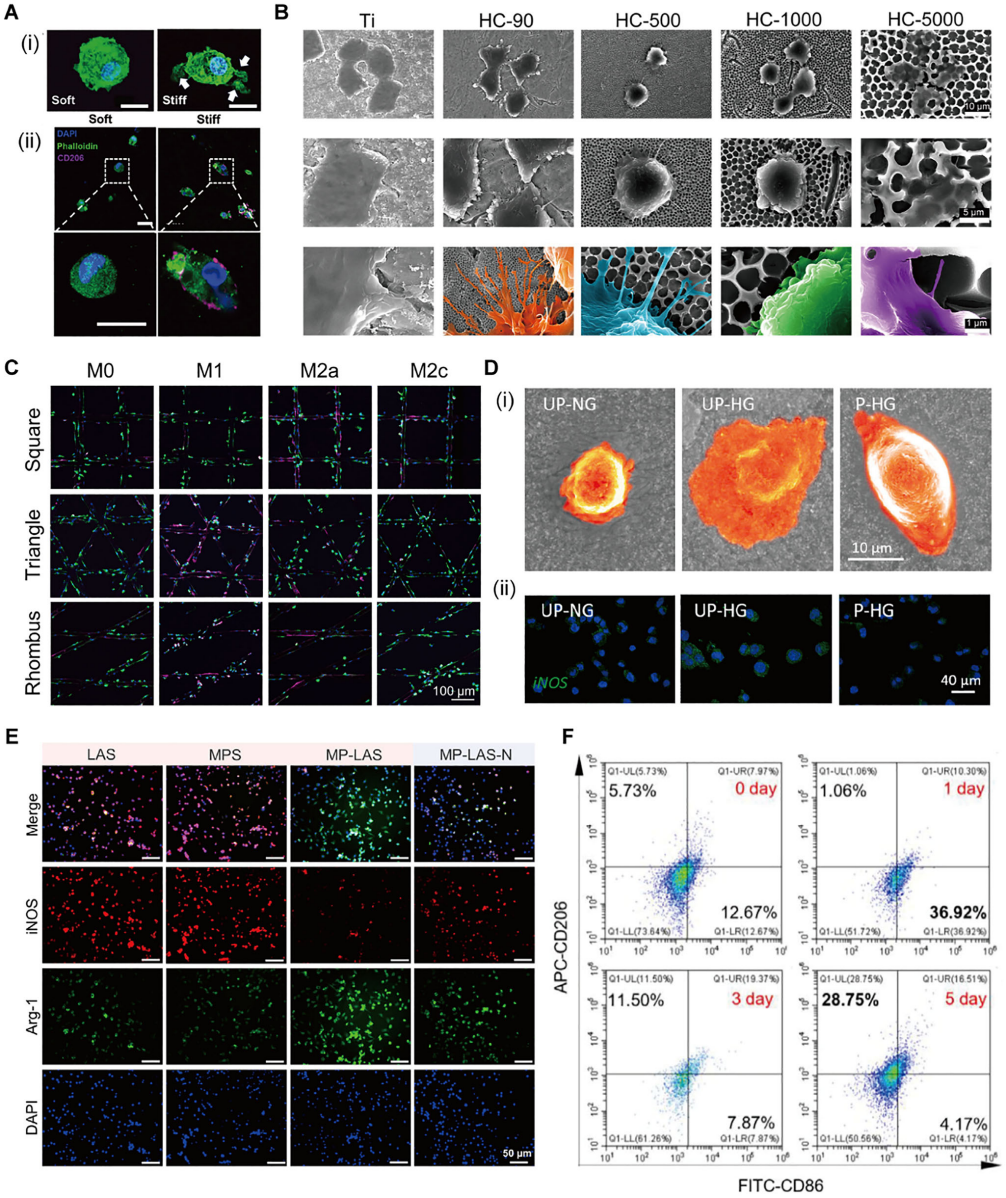
Research progress on strategies of macrophage polarization. (A) BMDM's phenotypic polarization upon dynamic stiffening. (i) Representative confocal images of BMDMs (green: phalloidin; blue: DAPI) encapsulated in the hydrogels for 2 days. (ii) Representative confocal images of BMDMs stained with CD206 (magenta), DAPI (blue), and phalloidin (green). Reproduced with permission [113] Copyright 2025, Wiley‐VCH GmbH. (B) Cell morphologies of macrophages on corresponding surface topography. Reproduced with permission [27] Copyright 2021, American Association for the Advancement of Science. (C) Immunofluorescence staining of macrophage surface markers CD86 and CD163 on different scaffold architectures. In the pictures, CD86 is shown in magenta, while CD163 is shown in green. Reproduced with permission [122] Copyright 2023, Elsevier. (D) Electrical microenvironment regulates phenotypic changes in high‐glucose‐induced macrophages. (i) Representative SEM images of macrophages cultured under different conditions. UP‐NG: unpolarized membranes cultured with normal glucose concentration; UP‐HG: unpolarized membranes cultured with high glucose concentration; P‐HG: polarized membranes cultured with high glucose concentration. (ii) Representative immunofluorescence images iNOS (green) and cell nuclei (DAPI, blue) in macrophages cultured for 24 h. Reproduced with permission [132] Copyright 2020, Elsevier. (E) Bioactive factors regulate macrophage polarization. Analysis of macrophage (Raw264.7) phenotypic markers using immunofluorescence staining. In the MP‐LAS group, NO is generated efficiently. Reproduced with permission [142] Copyright 2026, Wiley‐VCH. (F) Temporal release of Ca^2^^+^ and Zn^2^^+^ regulates macrophage polarization. FCM for detecting the expression of CD86 and CD206 on macrophage surfaces. Reproduced with permission [161] Copyright 2026, Wiley‐VCH.

However, Lorenzo Moroni and co‐workers drew the opposite conclusion. In their study, they chose two chemically relevant and biocompatible materials with virtually identical chemistries based on poly(lactide‐co‐caprolactone), one amorphous and another semicrystalline [112]. These materials had virtually identical chemical compositions, while differing in Young's modulus by two orders of magnitude (4 kPa vs 400 kPa). They found that scaffolds with higher mechanical properties led to an M2‐like macrophage polarization in vitro and to tissue formation and remodeling in vivo. In contrast, softer scaffolds led to an M1 polarization characterized by the production of a high amount of TNF‐𝛼. Analysis revealed that macrophages had a larger spread surface area on stiffer surfaces, promoting M1 subtype and inducing an anti‐inflammatory phenotype with a smaller spread area on softer surfaces. Another study also supports this opinion, showing that increasing matrix stiffness alone promoted macrophage polarization toward a pro‐regenerative phenotype (Figure 4A) [113]. Therefore, it is essential to consider other material properties such as the adsorption capacity of proteins (e.g., fibronectin and fibrinogen) and macrophage spread area on the substrate surface when designing biomaterials to regulate macrophage polarization, rather than adopting a simple guide theory to design material within some stiffness values to control the polarization of macrophages.

### 9.2 Surface Topography

Surface topography, such as roughness and oriented structure, can impact the fate of macrophages and cytokine secretion, thereby modulating the local immune environment and ultimately promoting bone regeneration [114, 115]. Studies have shown that macrophages have a preference for adhering to rough substrate surfaces compared to smooth ones [107]. For example, Zhu et al. conducted a study on titanium with different scales of honeycomb‐like structure TiO_2_ structures (90, 500, 1000, and 5000 nm) to activates the M2 phenotype in macrophages. They found that decreasing the size of micro‐structures could effectively activate the M2 phenotype, in which the 90‐nanometer sample showed the highest expression level of CD206, IL‐4, and IL‐10, as well as the highest release of bone morphogenetic protein‐2 compared to samples with larger scales (Figure 4B) [27]. Besides, through utilizing a micropatterning approach to directly control the shape of macrophages, researchers observed that the transduction of cell shape to changes in macrophage phenotype was dependent on contractility within the actin cytoskeleton [116, 117]. For example, micropatterns with a width of 20‐µm induced macrophage polarization toward the M2 phenotype, as evidenced by their elongated morphology [118]. In addition, Shuai et al. discovered that simply aligning the lamella implant parallel to the long axis of the bone can promote bone regeneration by modulating macrophage polarization [119].

### 9.3 Geometry of Scaffold

The geometry of implanted materials, including their size and shape, also has a significant impact on macrophage polarization [120, 121]. Recently, Mondadori et al. fabricated Polycaprolactone (PCL) scaffolds by the Melt Electrowriting strategy to investigate the ability of scaffold architecture to modulate macrophage polarization (Figure 4C). The research results showed that the rhombic structure promoted the release of IL‐10, IL‐13, and sCD163 by M2a macrophages [122]. The size of particles can affect macrophage phagocytosis, with larger particles being more difficult for macrophages to phagocytose. Materials with sharp edges, such as pentagons and triangles, can induce more severe inflammatory reactions compared to spherical particles [123, 124]. Moreover, compared to particles over 1 µm, nanoparticles have been shown to stimulate the secretion of IL‐1β in microphages, resulting in a more inflammatory response [125]. In addition, the use of microfibers made from various polymers (polyester, polyethylene, PLLA, and polyurethane) with differing diameters (1–5, 6–10, and 11–15 µm) showed that those with a diameter of 1‐5 µm elicited a minimal host response [126]. Therefore, when designing materials, it is important to consider the appropriate surface topography and geometric structures that can induce macrophages to polarize toward the M2 direction.

### 9.4 Surface Charge

The overall negative charge of macrophage cellular membranes makes the surface charge of biomaterials an important factor in modulating the macrophage response [127]. In general, positively charged (cationic) particles are more likely to trigger an inflammatory response than negatively charged (anionic) and neutral particles. Cationic polymers such as polylysine, polythylenimine (PEI), cationic gelatin, and dextran could activate M1 macrophages via the toll‐like receptor‐4 (TLR‐4) pathway and specifically induce IL‐12 secretion to strongly stimulate Th1 response. In contrast, certain other positively charged materials can promote the phenotypic shift of macrophages from the M1 state toward the M2 state. For example, the cationic carrier PAMAM suppresses inflammation by neutralizing cfDNA and facilitates the transition from M1 to M2 macrophages [128]. Similarly, berberine—a cationic alkaloid—also exhibits M2‐polarizing activity [129]. Additionally, positively charged particles are more readily internalized by macrophages compared to negatively charged or neutral particles [130]. For example, macrophages have higher uptake of positively charged liposomes compared with negatively charged liposomes [131]. There is also a study showing that the polarized BaTiO_3_/poly BTO/P(VDF‐TrFE) nanocomposite membranes inhibited high glucose‐induced M1‐type inflammation by effecting changes in cell morphology, M1 marker expression, and pro‐inflammatory cytokine secretion in macrophages (Figure 4D) [132]. Collectively, manipulating the surface charge of biomaterials offers a powerful strategy to modulate the immune response of macrophages to implanted biomaterials. This can be beneficial for purposes such as evading phagocytosis, regulating inflammation, and controlling the foreign body reaction.

## 10 Bioactive Release

Bioactive factors such as exogenous growth factors and bioactive ions can effectively modulate inflammatory behavior [133, 134, 135]. When designed in a proper delivery strategy, these factors equip the scaffolds with the capability to promote bone regeneration.

### 10.1 Bioactive Factors

The growth factors and cytokines can significantly affect the direction of macrophage polarization [136]. Considering that exogenous biological factors usually have short half‐lives and easily denatured or degraded. Therefore, appropriate materials are necessary to ensure the prolonged release of these factors [137]. For example, incorporating IL‐4 into nanofibrous heparin‐modified gelatin microspheres has been shown to achieve sustained release, effectively converting M1 to M2 macrophage phenotype and enhancing bone regeneration [138]. Besides, BMPs also are the candidate loading cytokines into bone scaffolds, which can achieve the recruitment of MSCs, regulation of macrophages, and osteoimmunomodulation under the inflammatory phase [86]. For example, Sun et al. prepared GelMA/gelatin/PEG scaffolds that composite mesoporous silica nanoparticles (MSNs) with loading BMP‐4, and they found that BMP‐4 can be sustainably release from MSNs and reduced the level of inflammation in calvarial‐bone defects by activating the polarization RAW264.7 to M2 macrophages [139].

In some cases, the simultaneous release of multiple factors can have a synergistic effect, leading to better regulation of inflammation and improved therapeutic efficacy in bone repair [140]. For example, an interpenetrating hydrogel network loaded with IL‐4 and BMP‐2 has been developed, which significantly facilitates M2 macrophage differentiation and osteogenic differentiation of bone marrow stromal cells, as well as reducing local inflammation and enhancing bone regeneration [141]. Guo et al. developed an MP‐LAS scaffold based on a microporous confinement strategy, which enables precise regulation of the ROS/L‐Arg reaction to achieve in situ NO generation. This system can maintain a stable NO supply over a period of 3 months and promote osteogenesis through mechanisms including the regulation of macrophage M2 polarization (Figure 4E) [142]. While the introduction of exogenous biological factors can be beneficial for macrophage polarization, caution should be exercised when using systemic factors due to their wide distribution of receptors, uncertainty regarding target actions, and potential immune responses that may lead to off‐target effects in vivo.

### 10.2 Ion‐Doping

Bioactive ions play a crucial role in regulating the secretion of pro‐inflammatory and anti‐inflammatory cytokines from cells, and in turn, enhance bone regeneration by modifying the local bone microenvironment. Calcium (Ca), for example, can reduce inflammatory responses by inhibiting the NF‐κB and Wnt5a/Ror2 signaling pathways [143, 144, 145]. Magnesium (Mg) can inhibit the activation of NF‐κB and promote the secretion of anti‐inflammatory cytokines, but controlling the release of Mg ions is a challenge due to the rapid degradation of Mg‐based materials [146, 147, 148, 149, 150, 151, 152]. Other bioactive ions such as Zinc (Zn) [153, 154, 155], strontium (Sr) [156, 157], copper (Cu) [158, 159], and cerium (Ce) [160] can also effectively modulate immune responses and improve bone regeneration. Recent studies have developed a temporal immunomodulatory hydrogel that releases Ca^2^^+^ in the early stage to promote M1 polarization for infection inhibition, while sustaining the release of Zn^2^^+^ to induce M2 polarization for osteogenic differentiation (Figure 4F) [161]. It is important to control the appropriate concentration range and release rate of bioactive ions to create a pro‐healing microenvironment while avoiding potential side effects and toxicity to normal tissues [162].

## 11 Strategies of Vascular Regeneration

**FIGURE 5:**
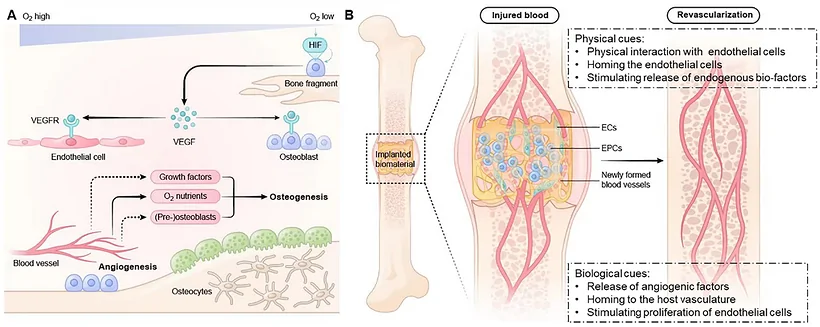
The bone regeneration through angiogenesis through various strategies. (A) The hypoxic conditions would activate the hypoxia‐inducible transcription factors (HIFs) signaling pathway, which plays a crucial role in regulating diverse biological processes, particularly the secretion of vascular endothelial growth factors (VEGFs). The pleiotropic effects of VEGF on cells involved in bone regeneration contribute to the promotion of angiogenesis/vascularization, osteogenic differentiation, etc. (B) Overview of the physicochemical properties and biological cues of bone scaffolds, including pore size, porosity, stiffness, bioactive metal ions and growth factors in promoting vascularization/angiogenesis and bone regeneration.

The oxygen tension in the surrounding environment plays a crucial role in regulating angiogenesis and vascularization [30, 64]. When oxygen levels are low, hypoxia‐inducible transcription factors (HIFs) become activated, allowing cells to survive and differentiate under low‐oxygen conditions. HIFs also stimulate the secretion of vascular endothelial growth factor (VEGF), which is a downstream target of the HIF pathway [66]. VEGF activates endothelial cells and osteoblasts, promoting angiogenesis and blood vessel formation. In addition to its role in angiogenesis, VEGF is also involved in the survival, activity, and recruitment of bone‐related cells, such as the differentiation of chondrocytes to hypertrophic, and mesenchymal progenitor cells differentiating into osteoblasts [65, 163, 164, 165]. Additionally, a hypoxic environment can promote the differentiation of mesenchymal precursor cells into chondrogenic lineages, and the chondrocytes are remarkably competent at surviving and differentiating in this hypoxic environment [61, 81]. The process of endochondral ossification is itself driven by vascularization as terminally differentiated chondrocytes (hypertrophic chondrocytes) induce an angiogenic switch that coordinates the breakdown of cartilage and its controlled replacement by vascularized bone tissue (Figure 5A) [166, 167].

Today, various strategies to achieve vascularization/angiogenesis in bone‐tissue engineering scaffolds have been explored using physicochemical cues, external stimulation, and biological cues (Figure 5B) [63, 168, 169, 170, 171].

## 12 Physicochemical Cues

Physicochemical cues play a significant role in regulating the behavior of endothelial cells, including their migration, sprouting, proliferation, and differentiation. The mechanical and physical signals from implanted materials can be converted into biochemical signals that influence these cellular activities. This approach has gained attention in recent years as it offers advantages over using external bioactive factors.

### 12.1 Porosity and Pore Size

**FIGURE 6:**
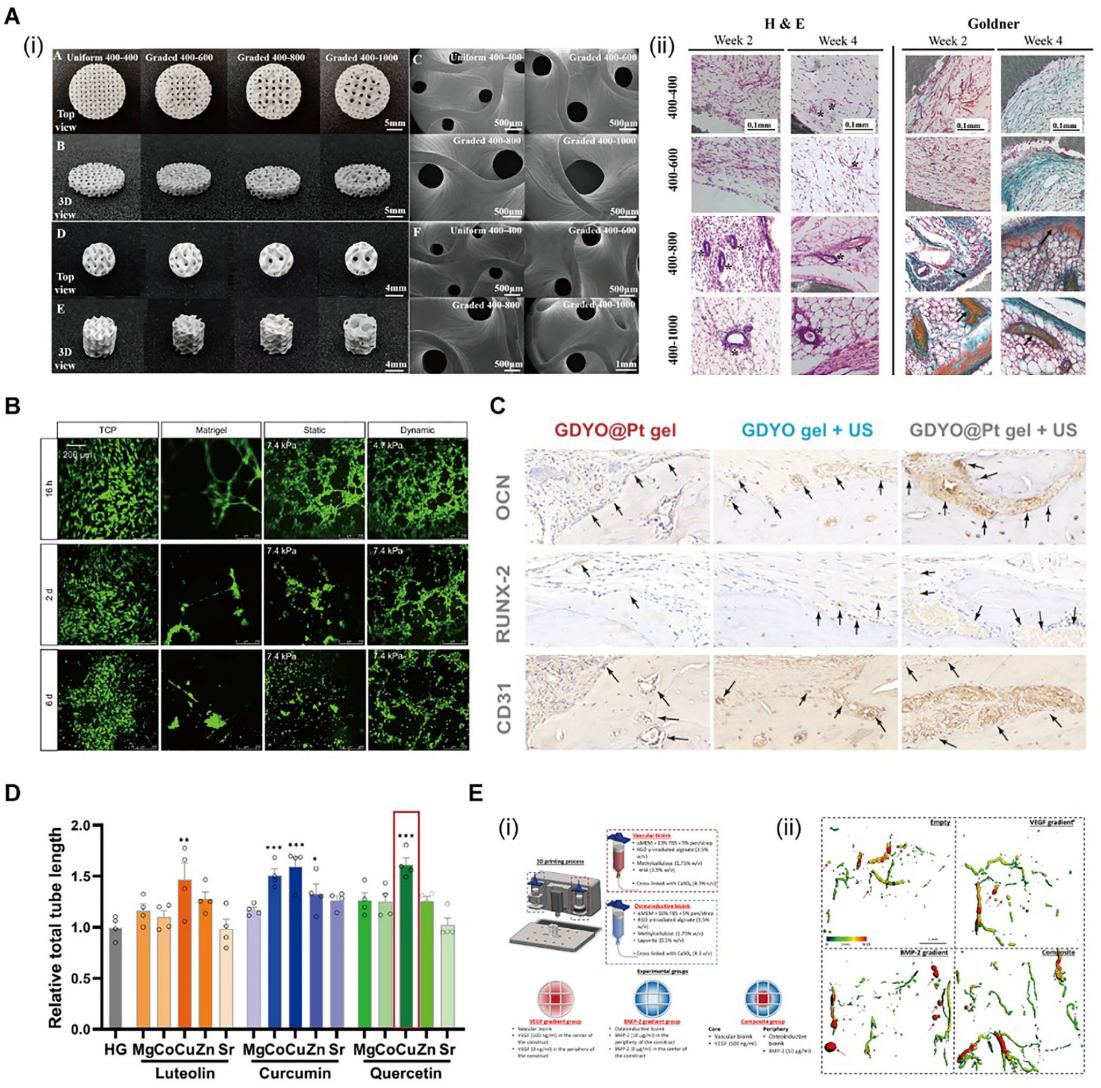
Research progress on strategies of vascular regeneration. (A) Angiogenic capacity of 3D‐printed scaffolds with different pore sizes. (i) Morphological characteristics of uniform pore size and PSG BCP scaffolds. (ii) Histological analysis of uniform pore size 400–400 scaffolds and graded 400–600, 400–800, and 400–1000 scaffolds after 2‐ and 4‐week subcutaneous implantation in mice. (black star: blood vessels). Reproduced with permission [179] Copyright 2025, Elsevier. (B) Angiogenic capacity of hydrogels with varying stiffness. Tube formation of HUVECs on TCP, Matrigel, static, and dynamic HAT‐Lap hydrogels at different time points demonstrating prolonged tube structure maintenance on the dynamic hydrogel. Reproduced with permission [184] Copyright 2025, Elsevier. (C) Piezoelectric gels promote angiogenesis and osteogenesis following ultrasound intervention. Typical immunohistochemical staining patterns demonstrating OCN, RUNX‐2, and CD31 expression in neoformed calvarial regions at 12 weeks post‐implantation. Reproduced with permission [192] Copyright 2026, Springer Nature. (D) The addition of metal ions promotes angiogenesis. Total tube length formed by HUVECs cultured with different metal–flavonoid‐coated β‐TCP scaffolds under HG conditions. Reproduced with permission [213] Copyright 2026, Wiley‐VCH GmbH. (E) Spatiotemporal delivery of both VEGF and BMP‐2 led to enhanced angiogenesis. (i) Schematic of the 3D printed experimental groups including key features of the developed bioinks and the segmental defect procedure. (ii) µCT angiography representative images of vessel diameter. Reproduced with permission [170] Copyright 2020, American Association for the Advancement of Science.

One important aspect of physicochemical cues is the porosity and pore size of scaffolds. These factors impact endothelial cell penetration, migration, nutrient diffusion, metabolic waste removal, and provide a three‐dimensional microenvironment that promotes tissue invasion and the formation of functional blood vessels [172, 173]. To promote angiogenesis, various fabrication techniques such as 3D printing, electrospinning, and freeze casting are used to regulate scaffold porosity [63, 174]. Typically, macropore sizes exceeding 300 µm combined with porosity levels greater than 50%–70% are conducive to vascularization; however, the optimal parameter range varies depending on factors such as material type and animal models. The presence of large pores and high porosities benefits endothelial cell adhesion, growth, and infiltration [175, 176]. Considering that blood vessel growth also involves nutrient diffusion and removal of metabolic substances, it is reasonable to design a scaffold with hierarchical structures [177]. For example, interconnected porous β‐tricalcium phosphate (β‐TCP) scaffolds with varying geometric channels have been developed and shown to enhance angiogenesis by promoting the infiltration, migration, and proliferation of endothelial cells [168]. A study conducted by Xiong et al. found that among HA scaffolds with pore sizes of 400/600/800 micrometers, the 600‐micrometer pore size more effectively improved the long‐term inflammatory response and promoted vascular proliferation and bone tissue formation [178]. Wang et al. also demonstrated that pore‐size graded (PSG) scaffolds possess better angiogenic potential than scaffolds with uniform pore sizes (Figure 6A) [179]. Future research may focus on designing hierarchical scaffolds with a reasonable gradient change in pore size and porosity to further enhance the blood vessel growth process. This dynamic approach recognizes the evolving nature of blood vessel formation and aims to create scaffolds that can provide an optimal microenvironment for angiogenesis.

### 12.2 Stiffness

Stiffness is a crucial biophysical property of materials that can have a significant impact on cellular behavior and function. Cells cultured on a scaffold with a specific stiffness respond to external mechanical cues through various signaling pathways mediated by membrane proteins like integrins. This response can trigger processes such as cell adhesion, proliferation, migration, and differentiation [180, 181]. Research has shown that the stiffness of a scaffold can influence cell infiltration and angiogenesis. For example, studies conducted by Lv et al. demonstrated that bone matrix scaffolds with low compressive modulus significantly enhanced cell infiltration and angiogenesis compared to scaffolds with high or medium modulus [182]. Another study by Wu and colleagues investigated the seeding of endothelial cells on materials with different stiffness. They found that well‐defined vascular networks formed on soft gels (11 to 36 kPa), but as the rigidity of the scaffolds increased, the formation of blood vessels reduced significantly [183]. Bu et al. present a dynamic hydrogel platform that imitates the progressive mechanical changes of the native extracellular matrix (ECM), transitioning from soft (0.8 kPa) to stiff (7.4 kPa) over 48 h. The gradually stiffening hydrogel promotes vascularized bone regeneration in a critical‐size cranial defect model (Figure 6B) [184]. These findings indicate that simply using biomimetic scaffolds that mimic mechanical properties may not be sufficient to promote angiogenesis. It is still not fully understood how cells perceive and translate mechanical signals into biochemical mechanisms, which ultimately initiate molecular processes. Further research is needed to gain a better understanding of these cellular processes and to design scaffolds that effectively translate mechanical cues into biochemical responses for angiogenesis.

## 13 External Stimulation

Among external strategies for promoting bone regeneration vascularization, electrical and magnetic field stimulation have been demonstrated to exhibit significant therapeutic efficacy [185, 186]. Electrical stimulation, including direct current fields, capacitively coupled fields, and endogenous fields arising from implantable electronics, has been demonstrated to promote vascularization [187]. Pulsed electromagnetic fields (PEMF), as a non‐invasive modality approved by the FDA for fracture healing, enhance neovascularization by upregulating pro‐angiogenic factors such as VEGF and HIF‐1α, thereby promoting endothelial cell proliferation, migration, and lumen formation, ultimately accelerating the establishment of nascent vascular networks [188]. Piezoelectric materials have emerged as promising candidates in orthopedics and bone regeneration due to their ability to mimic the natural electromechanical properties of bone and convert mechanical stimuli into electrical signals that promote bone repair [189, 190]. For instance, researchers have introduced a self‐reinforcing piezoelectric chip that generates self‐sustaining electrical signals through physiological vibrations, promoting vascularized bone repair [191]. Another study shows that ultrasound‐activated piezoelectric GDYO@Pt promotes angiogenesis and osteogenesis, accelerating cranial bone defect repair (Figure 6C) [192]. In a recent study, a biodegradable piezoelectric cryogel scaffold (PWH Gel) was developed to couple electrical and ionic cues under physiological loading, establishing a self‐powered microenvironment that synergistically promotes osteogenesis and angiogenesis for functional reconstruction of segmental bone defects [193]. The mechanisms underlying magnetic field–promoted angiogenesis have attracted increasing attention. A study showed that a 3D‐printed PLGA/γ‐Fe_2_O scaffold promoted angiogenesis in vitro and in vivo by inducing, via a static magnetic field, the upregulation of NF‐κB and HIF‐1α in endothelial cells, leading to increased VEGF expression and angiogenic activation [194]. Despite these encouraging results, optimization of stimulation parameters (intensity, frequency, and duration), mitigation of potential tissue heating, and advancement of clinical translation remain imperative. Overall, physical stimulation represents a drug‐free, remotely controllable platform that, when integrated with material‐based immunomodulation and cellular directive strategies, provides a comprehensive solution for vascularized bone tissue engineering.

## 14 Biologic Cues

### 14.1 Ion‐Doped Materials

Biologic cues in the form of ion‐doped materials and growth factors play critical roles in promoting vascularized bone regeneration. Bioinorganic ions, such as copper ions, cerium ions, strontium ions, iron ions, and magnesium ions, have been proven to promote vascularized bone regeneration by stimulating the formation of angiogenesis‐related and osteogenesis‐related growth factors [195, 196, 197, 198]. However, the direct use of these bioactive ions can lead to cytotoxicity and the release of high‐concentration metal ions, potentially causing secondary infections. To address this, bioactive ions are typically doped into suitable delivery systems such as scaffolds and particles to control their release and prevent complications [175, 199, 200, 201]. For example, magnesium (Mg^2+^)‐doped bone tissue scaffolds have gained attention due to their ability to regulate bone metabolism, promote new bone formation, and play a role in vascular growth and function [195, 202, 203, 204, 205, 206, 207]. Similarly, copper ions stimulate the proliferation of endothelial cells, dermal fibroblasts, and arterial smooth muscle cells, enhancing angiogenesis and bone regeneration through the involvement of various transcription factors and the release of growth factors and cytokines (Figure 6D) [199, 208, 209, 210, 211, 212, 213]. However, challenges remain in terms of interfacial bonding and interface compatibility between the material matrix and metal ions, as well as the need to control the release behavior of metals from scaffolds to avoid negative effects on cells and organs [177, 205].

### 14.2 Bioactive Factor

During vascular bone regeneration, growth factors and cytokines (e.g., VEGF and PDGF) play a vital role in the process of bone regeneration by impacting the activation, migration, and differentiation of ECs and OBs/OCs. However, the inherent short biological half‐life and high instability of these growth factors limit their efficacy and clinical application. To address these limitations, researchers are developing delivery systems that enable spatiotemporal control of growth factor release [214, 215, 216, 217, 218, 219]. For example, Freeman et al. designed bio‐scaffolds with concentration gradient variation of VEGF and BMP‐2 to regulate release them in space and time. Compared to controls homogenously loaded with the same total amount of growth factor, the designed scaffolds with spatiotemporal releasing character significantly enhanced angiogenesis and bone formation, and accelerated large bone defect healing (Figure 6E) [170]. However, the effectiveness of exogenous growth factors in clinical applications must be further investigated due to their short half‐life and potential immune responses. Therefore, the development of effective delivery systems to regulate the release of these biological factors remains a focus of research.

## 15 Strategies of Nerve Regulation

**FIGURE 7:**
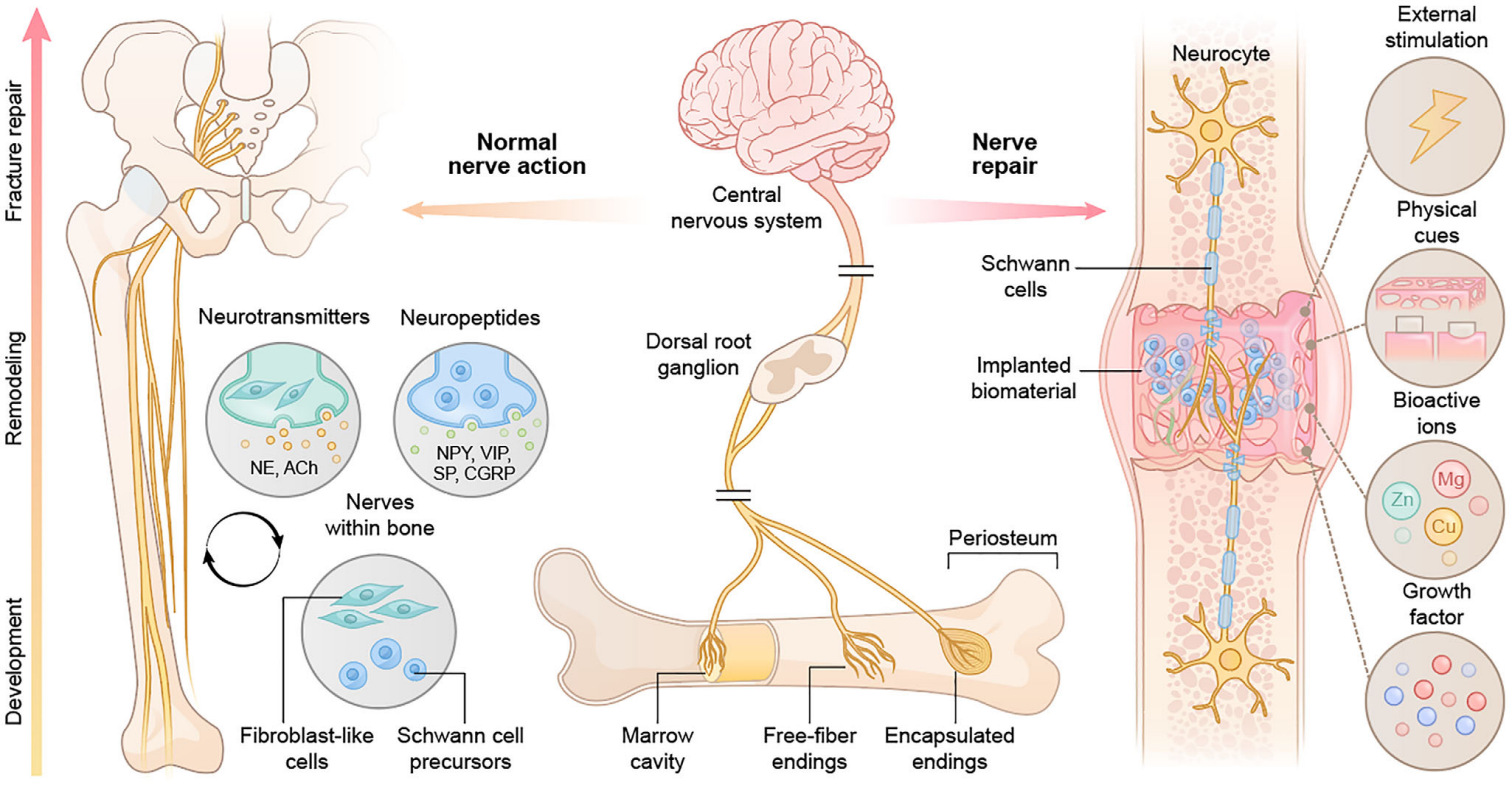
(A) Bidirectional communication between nerves and bone within the skeleton. For bone development, bone remodeling, and bone regeneration, the nerve cells, together with the released neurotransmitters and neuropeptides, play important regulatory roles in bone metabolism. NE, norepinephrine; ACh, acetylcholine; NPY, neuropeptide Y; VIP, vasoactive intestinal peptide; SP, substance P; CGRP, calcitonin gene‐related peptide. (B) Designing biomaterials with physical, biological, and electrical cues to mediate the activities of nerve cells to promote bone regeneration.

Nerves are present throughout various types of bone, with a dense network at the periosteum and accompanying nutritive vessels in the bone marrow and Haversian system. These nerves interact with the skeleton to regulate bone metabolism through nerve‐resident cells, locally released neurotransmitters, neuropeptides, axon guidance factors, and neurotrophins (Figure 7A).

The restoration of nerve supply is crucial for bone regeneration during the healing process. Intrabony nerves play a role in regulating blood regeneration and stem cell behavior through neurotransmitters, such as calcitonin gene‐related peptide (CGRP). Blocking neuropeptides like CGRP can hinder bone repair. Deficiencies in sensory or sympathetic nerves can also lead to inferior bone healing [220]. In recent years, neurotization has emerged as a promising strategy in bone tissue engineering to address the challenge of treating larger‐sized bone defects (Figure 7B) [72, 78, 221]. Neurotization involves incorporating nerve supply into tissue‐engineered scaffolds to enhance bone regeneration. However, it's important to note that research in the field of neuralized bone tissue engineering is still at an early stage of development and relatively obscure. Several reviews have provided brief overviews and valuable perspectives on neuralized bone tissue engineering, making them useful resources for those interested in exploring this emerging field further. A recent study demonstrates that sensory nerves innervating bone actively regulate fracture healing, rather than functioning solely as pain‐transmitting units [222]. Single‐cell transcriptomic analyses reveal pronounced stage‐specific changes in the neural transcriptome following fracture: the early phase is characterized by the enrichment of pain and inflammation‐associated genes, whereas the repair phase features the upregulation of regeneration‐related signaling pathways. Furthermore, sensory neuron‐derived FGF9 is identified as a key mediator of bone regeneration via FGFR signaling. Collectively, these findings suggest that future bone repair biomaterials should be designed not only to promote osteogenesis but also to preserve or recapitulate neurotrophic and neuro‐paracrine cues in a temporally regulated manner.

## 16 External Stimulation

**FIGURE 8:**
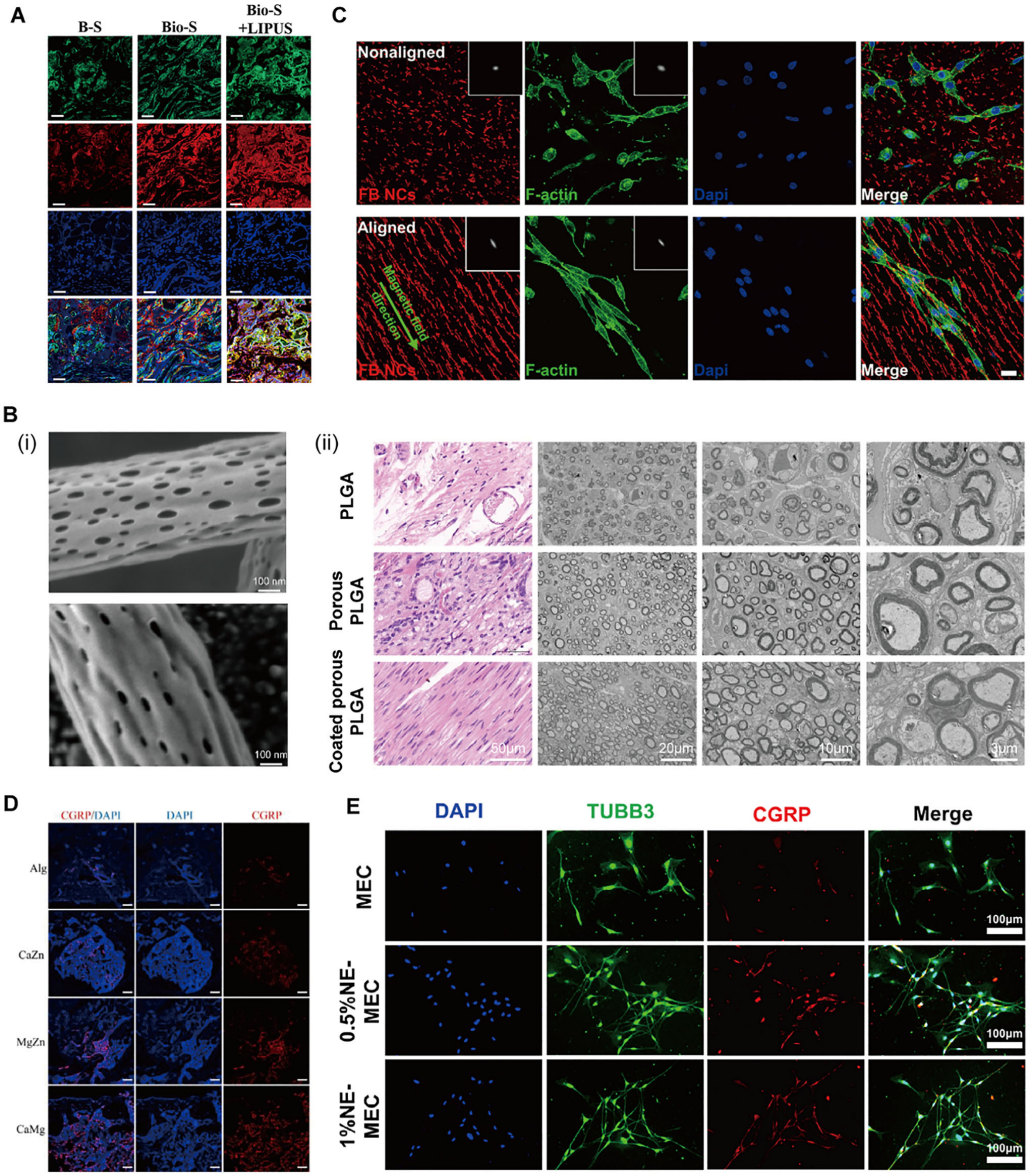
Research progress on strategies of nerve regulation. (A) Ultrasonic‐driven wireless electrical stimulation facilitated neuro‐vascularization. Representative immunofluorescence images of CD31 (green) and CGRP (red) in the defect areas after 8 weeks of implantation. Reproduced with permission [232] Copyright 2024, Elsevier. (B) Porous Fibrous Conduits Promote Nerve Regeneration. (i) The surface morphology of uncoated and coated porous PLGA fiber films. (ii) The regenerated nerves in each group were evaluated at 3 months after treatment using H&E staining and TEM images. Reproduced with permission [238] Copyright 2024, Elsevier. (C) The aligned hydrogel topography induces cells to orient along the fiber direction. Confocal images of PC12 cells in nonaligned and aligned 3D magnetoelectric hydrogels. Reproduced with permission [249] Copyright 2023, Elsevier. (D) Metal ions facilitate neurogenesis. Representative immunofluorescence images showing CGRP‐positive area in the bone defects with treatment by the blank Alg or the dual metal cations. Reproduced with permission [253] Copyright 2025, Elsevier. (E) The incorporation of Norepinephrine (NE) enhances neurogenesis. TUBB3 and CGRP staining of DRG neurons cultured with conditioned media at day 6. Reproduced with permission [263] Copyright 2026, Wiley‐VCH GmbH.

Electrical signals not only serve as a means of communication between neurons but also play a role in regulating nerve growth and other neural physiological activities. For instance, electrical stimulation can increase nerve fiber density and upregulate the expression of nerve‐related biological factors [223]. In recent years, electric stimulation has been used to guide the behavior of stem cell‐derived neural cells, promoting bone regeneration [224]. Researchers have implanted soft electrodes at dorsal root ganglia, which stimulate the biosynthesis and release of calcitonin gene‐related peptide (CGRP), leading to improved healing of femoral defects [225]. However, most electrical circuits are designed for short‐term healing, which may cause side effects such as bacterial contamination, scarring, and chronic inflammation in long‐standing wounds. While minimally invasive or non‐invasive strategies like magneto‐electric or endogenous electric field stimulation have been proposed, finding a suitable electrical stimulation technique remains a challenge for nerve regeneration in injured bone tissue [226, 227, 228, 229]. Recent studies have also shown that pulsed electromagnetic fields (PEMFs) promote osteogenic differentiation by activating sensory nerves within bone, stimulating their secretion of Sema3A, which then acts through the Nrp1/Plexin‐A1 receptors on the surface of Mesenchymal Stem Cells (MSCs). Piezoelectric materials can also promote nerve regeneration during the bone repair process. A study demonstrated that an injectable, ultrasound‐responsive piezoelectric conductive fiber network regionally activates Schwann cell‐derived exosomal miRNAs, thereby promoting neurogenic bone regeneration via enhanced neural network formation and osteogenic differentiation [230]. The researchers developed a bioceramic scaffold coated with a polylactic acid (PLA) nanofiber membrane (TCP‐PLA/GeSe). By enabling spatiotemporal control of piezoelectric stimulation under ultrasound, this biomimetic scaffold regionally activates neurogenic differentiation of Schwann cells via intracellular Ca^2^^+^ elevation and the activation of PI3K‐Akt/Ras signaling pathways, thereby promoting early innervation to support neurogenic bone regeneration [231]. Zhao et al. show that a bioinspired bilayer scaffold significantly enhances osteogenesis, angiogenesis, and neurogenesis combined with low‐intensity pulsed ultrasound‐assisted piezoelectric stimulation (Figure 8A) [232]. This has renewed attention on how external stimuli can promote bone formation through neural regulation [233].

## 17 Physical Cues

### 17.1 Porous

Porous scaffolds can mimic the microenvironment of the extracellular matrix, providing an essential physiological environment for cell adhesion, migration, and proliferation. Therefore, it is crucial to design scaffolds with suitable pore architecture, including pore size, porosity, pore interconnectivity, and wall thickness (Figure 8B) [78, 220, 234, 235, 236, 237, 238]. For example, high porosity (> 80%) is desirable to ensure efficient exchange and transportation of oxygen, nutrients, and waste tissues. Hierarchical pore architectures in scaffolds offer a larger surface area for functionalization, cell adhesion, and migration compared to scaffold designs with a single pore size [239, 240]. Besides, scaffolds with hierarchical pore architectures offer superior surface area for functionalization and cell adhesion and migration as compared to a single‐size aperture. Typically, nanopores and micropores (< 10 µm) are suitable for protein absorption and cell adhesion. Micropores between 10 and 50 µm are beneficial for forming fibrous tissue, while macropores larger than 150 µm can support the migration of nerve cells into the inner core of implants [241, 242, 243]. However, increasing pore architectures may compromise the mechanical properties of the implants, especially compression performance. Thus, the porous properties of scaffolds must be carefully designed to meet the mechanical and permeability requirements for nerve‐guided bone regeneration [241, 244].

### 17.2 Topography

It is generally considered that a matrix with highly aligned characteristics can stimulate Schwann cell orientation and axon growth, thereby facilitating regeneration of injured nerve [245, 246, 247]. As an example, Wang et al. designed a 3D scaffold featuring both aligned nanofibers and aligned interconnected macro‐channel to mimic the extracellular matrix of anisotropic tissues. Such aligned structures closely resembled natural nerve conduits and provided superior support for nerve regeneration [237]. Besides, scaffolds that combine aligned multi‐channels with hierarchical pore architectures have shown promise in inducing nerve‐guided bone regeneration [241]. For example, Chen et al. created a 3D porous scaffold with a nano‐topology resembling a “coral reef‐like” rough surface using a freezing casting strategy. This scaffold, with its hierarchical structures, promoted neurite outgrowth and upregulated synaptophysin expression in neuron‐like cells (PC12), facilitating the regeneration of both nerve and bone tissues [248]. Furthermore, research has integrated wireless electrical signals with topological cues, thereby facilitating the growth of orderly aligned neural cells (Figure 8C) [249].

## 18 Releasing Bioactive Components

### 18.1 Ion‐Doped Materials

Human bones are composed of various elements, such as calcium, magnesium, phosphorus, and zinc, which directly affect the growth and regeneration of nerves. For example, calcium, as the main inorganic component of bone, significantly impacts the regeneration of nerves and bones by regulating corresponding growth factors for both tissues [250]. Phosphorus also plays a key role in regulating the proliferation and differentiation of neural stem cells (NSCs) [251, 252]. The divalent cations (Mg^2+^, Zn^2+^, and Ca^2+^) significantly upregulated levels of sensory nerve‐derived CGRP and PNS‐produced choline acetyltransferase, while significantly tuned down sympathetic activity in the defect zone in rats, thereby contributing to significantly increased bone formation [253] (Figure 8D). Besides, other trace elements, e.g., silicon (Si), strontium (Sr), and cobalt (Co), have the ability to regulate neurogenesis and promote bone healing. For example, Si ions not only induce osteogenic differentiation of bone marrow mesenchymal stem cells (BMSCs) but also enhance axonal growth and stimulate the secretion of neuropeptides [254]. Implants containing magnesium have also shown effectiveness in bone fracture repair, partly due to magnesium ions promoting the production of neuronal calcitonin gene‐related polypeptide‐alpha (CGRP) [255, 256]. Yu et al. constructed a calcium titanate (CaTiO3) coating with micro/nano‐topography, effectively promoting neurovascular osseointegration of titanium alloy scaffolds [257]. Therefore, incorporating therapeutic ions into implanted biomaterials for controlled release in the injury area has become a research focus in order to mitigate these issues, as discussed in the previous section.

### 18.2 Bioactive Factor

Loading of scaffolds with biological factors such as growth factors and peptides (e.g., NGF, neuropeptide Y (NPY), substance P (SP), and CGRP, etc.) can effectively promote osteogenesis and/or neurogenesis. When properly designed, these factors confer neuro‐attractive capabilities to the scaffolds, facilitating nerve regeneration. For example, incorporating NGF into the scaffold can achieve the regenerating nerval axons as well as nerve‐mediated bone regeneration [258, 259, 260, 261]. Xu et al. innovatively incorporated a sensory nerve‐homing peptide into the hydrogel, selectively enhancing endogenous sensory innervation and effectively promoting bone regeneration through immune modulation [262]. Norepinephrine was found to enhance the osteoinductive efficacy of the biomimetic mineralized scaffold, an effect that was associated with an upregulation of nerve growth factor expression and CGRP production (Figure 8E) [263]. Moreover, the scaffold with related cells is another commonly used strategy. For example, Thrivikraman et al. prepared a biomimetic matrix by culturing the undifferentiated human mesenchymal stem cells (hMSCs) in supersaturated calcium and phosphate‐rich cell media, which can ensure the formation of nanoscale hydroxyapatite into the interstices of collagen fibrils; such a bio‐scaffold can stimulate the osteogenic differentiation of hMSCs and the formation of blood capillaries and neuronal networks [223]. These biological factors do have the ability to promote neurogenesis and osteogenesis. However, their effectiveness is hindered by their short biological half‐life and high instability, which greatly restricts their practical application. Therefore, great efforts should be devoted to the development of effective delivery systems to regulate the release of these biological factors, as discussed in the previous section.

In brief, the healing of bone fractures is a spatiotemporal coordination process in which neural, vascular, and bone repair mechanisms work synergistically. From a spatial perspective, newly formed nerves and blood vessels are regulated by complementary guidance cues and often grow along similar pathways into the fracture callus [264]. Temporally, during the hematoma phase of fracture repair, nerves regulate local blood flow through vasomotor modulation, which is crucial for hematoma formation and stability, as well as for the recruitment and activation of immune cells [265, 266]. Upon entering the callus phase, nerves and blood vessels regenerate and invade the bone tissue to restore normal innervation and blood circulation, thereby replacing necrotic bone. Some experimental fracture models demonstrate that signals of nerve fiber regeneration in the callus region appear earlier than those of vascularization and osteogenesis‐related markers, suggesting that reinnervation may initiate or regulate subsequent angiogenesis and bone formation [267], while the vascular and immune microenvironment, in turn, influences nerve invasion and maintenance [268]. Impairment of nerve reinnervation is often accompanied by reduced vascular density and weakened osteogenic capacity, thereby delaying bone defect healing. In rodent models, inhibition of the TrkA signaling pathway leads to delayed vascular invasion of primary and secondary ossification centers, further reducing femoral length and volume [269, 270]. In the future, materials design should consider the spatiotemporal interactions among neural, vascular, and bone repair to enhance bone repair.

## 19 Conclusion and Perspective

**FIGURE 9:**
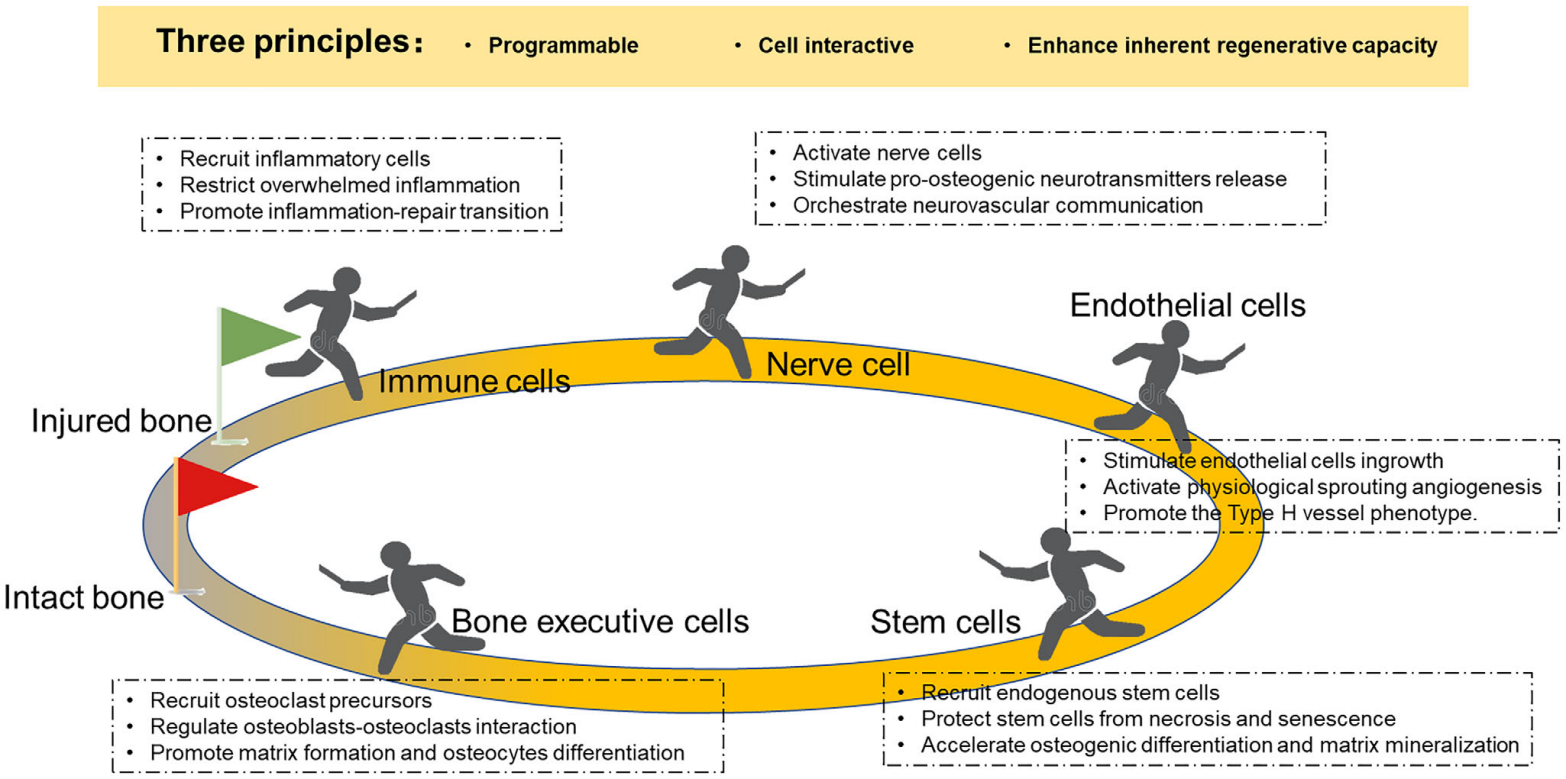
Optimal material design should be like the runway and sports gear to the athletes.

The process of bone repair can be likened to a relay race, with programmable bio‐interactive scaffolds acting as the relay athletes, as depicted in Figure 9. First, they should possess the capability to regulate the behavior of related cells involved in bone repair. This includes functions such as cell homing, adhesion, proliferation, and differentiation, which can be controlled by the materials themselves without relying solely on the incorporation of exogenous bioactive components.

Second, since bone regeneration is a dynamic process involving interactions between cells and the surrounding matrix, it is crucial to use programmable bio‐interactive scaffolds that can adapt to the changing cellular environment. These scaffolds should have the ability to alter their physicochemical properties in response to biological signals, ensuring the efficient completion of each repair phase and enabling timely initiation of subsequent stages. However, there are several challenges that need to be addressed in this field of research. (1) There is a limited understanding of the mechanisms of action of most biomaterials and their cellular response. Decoupling different material properties is difficult using current design strategies, making it challenging to independently alter each property and identify specific effects. Therefore, it is necessary to develop suitable processing strategies that allow for the regulation of material properties in a mono‐variable manner. (2) Conflicting requirements need to be addressed in material design. For example, mechanical stability is desired in bone repair, but rapid degradation is also needed for accelerated tissue ingrowth. Composite materials offer potential solutions by combining contradictory properties, but different material types often require different processing conditions, which complicates the design of biomaterial systems. (3) Bone regeneration is a dynamic process that involves multiple stages of repair. Currently, there is a lack of bio‐interactive materials that can adapt to local cellular changes and alter their physicochemical properties in response to biological signals at different repair stages. (4) While single‐cell sequencing has provided valuable insights into the genetic‐level interactions between materials and cells, further validation is needed from diverse perspectives. For example, incorporating appropriate gene knockout experiments in future bone regeneration research is essential for more rigorous investigations. (5) mRNA‐LNP therapy has gained extensive research attention in areas such as cancer treatment [271, 272, 273]. Although researchers have attempted to develop bone‐targeted LNPs [274, 275, 276], the application of mRNA‐LNP therapy in bone repair remains relatively limited due to the scarcity of related studies and the relatively simplistic existing bone‐targeting designs. As a highly promising and translationally favorable delivery platform, LNP may emerge as a novel therapeutic approach for bone repair in the future. Furthermore, the use of polymeric materials to load and sustain the release of multiple mRNA‐LNPs may enable the sequential regulation of neurogenesis, angiogenesis, and osteogenesis, thereby promoting bone defect healing.

Lastly, the accelerating global aging population underscores the critical need for breakthroughs in repairing osteoporotic bone defects. While traditional biomaterials offer basic mechanical support and osteoconductivity, their efficacy is limited by the pathological bone microenvironment—characterized by reduced osteoblast activity, heightened osteoclast function, persistent low‐grade inflammation, and impaired angiogenesis. To overcome barriers in osteoporosis therapy, we propose two integrated research directions. First, a multi‐target material design paradigm aims to rebalance bone remodeling by synergistically modulating the immune environment (e.g., polarizing M1 to M2 macrophages), inhibiting bone resorption (e.g., via bisphosphonates or gene editing), and promoting osteogenesis (e.g., through topological cues and growth factor release). Second, an adaptive innovation system for clinical translation focuses on developing injectable, fast‐setting biomaterials and additive manufacturing for patient‐specific fabrication, alongside establishing standardized ageing animal models to ensure a robust pipeline for efficacy and safety evaluation.

## Author Contributions

**Licheng Zhang**: conceptualization, supervision, funding acquisition. **Pengbin Yin**: supervision, conceptualization. **Jianjun Wang**: conceptualization, supervision, funding acquisition. **Fuming Cao**: writing – original draft, writing – review and editing. **Qingrui Fan**: writing – original draft, writing – review and editing. **Junqiang Mao**: investigation. **Peifu Tang**: conceptualization, funding acquisition.

## Conflicts of Interest

The authors declare no conflicts of interest.

## Data Availability

The data that support the findings of this study are available from the corresponding author upon reasonable request.
